# Hydatid cyst fluid promotes peri-cystic fibrosis in cystic echinococcosis by suppressing miR-19 expression

**DOI:** 10.1186/s13071-016-1562-x

**Published:** 2016-05-13

**Authors:** Chuanshan Zhang, Limin Wang, Tuergan Ali, Liang Li, Xiaojuan Bi, Junhua Wang, Guodong Lü, Yingmei Shao, Dominique A. Vuitton, Hao Wen, Renyong Lin

**Affiliations:** Xinjiang Key Laboratory of Echinococcosis, Clinical Medicine Institute, The First Affiliated Hospital of Xinjiang Medical University, Urumqi, Xinjiang China; State Key Laboratory Incubation Base of Xinjiang Major Diseases Research (2010DS890298), The First Affiliated Hospital of Xinjiang Medical University, Urumqi, Xinjiang China; WHO-Collaborating Centre for the Prevention and Treatment of Human Echinococcosis, Department of Parasitology, University of Franche-Comté (EA 3181) and University Hospital, Besançon, France

**Keywords:** *Echinococcus granulosus*, Liver fibrosis, HSC, miR-19, TβRII

## Abstract

**Background:**

*Echinococcus granulosus* infection causes cystic echinococcosis (CE); the generation of liver fibrosis around the parasitic larval cyst (metacestode) may play a major role in the spontaneous limitation of the parasitic growth; however, fibrogenesis has received little attention in CE. It has been reported that miR-19b plays a role in various diseases, including infectious diseases, by regulating fibrogenesis. However, its function in the development of liver fibrosis in *E. granulosus* infection is unknown.

**Methods:**

The expression of miR-19b and genes that are involved in liver fibrosis were analysed in *E. granulosus*-infected human livers using qRT-PCR. The role of miR-19b on hepatic stellate cells (LX-2 cells in vitro) treated with hydatid cyst fluid (HCF) was then analysed by 3-(4, 5-dimet-hylthiazol-2-yl)-2, 4-diphenyl-tetrazolium bromide (MTT) assay, qRT-PCR, Western blot and flow cytometry.

**Results:**

The results showed that the expression of miR-19 was significantly reduced in the pericystic collagen-rich liver tissue of CE patients, compared to normal liver. Incubation of LX-2 cells (in vitro) with HCF induced a decreased proliferation of these cells and a reduced expression of miR-19, inversely correlated with the expression of collagen 1A1 and TGF-β receptor II (TβRII). Conversely, overexpression of miR-19 by LX-2 cells inhibited the proliferation of these cells and led to decreased TβRII expression.

**Conclusions:**

Our study provides new evidence for the intervention of miRNAs in the regulation of fibrosis in infectious diseases; it suggests that *E. granulosus* can inhibit miR-19 liver expression and promote fibrosis through the increase in TβRII, the activation of hepatic stellate cells and extracellular matrix production.

## Background

Cystic echinococcosis (CE) is a chronic helminthic disease caused by infection with the metacestode (larval stage) of the tapeworm *Echinococcus granulosus*, one of the most widespread zoonotic diseases in humans in both developing and developed countries. About four million people are infected and another 40 million people are at risk [1–3]. The cyst which characterizes CE in intermediate hosts grows slowly. From inside to outside, it consists of an inner germinal layer and a peripheral laminated layer. A fibrotic pericyst is the result of a transient granulomatous reaction from the host’s immune system. The cyst is filled with hydatid cyst fluid (HCF) [4]. HCF results from the secretion of soluble components by the germinal layer of the metacestode and may also provide nutrients and other components necessary for the growth and development of the larval cyst and of the protoscoleces that are collected within the HCF; these protoscoleces will generate the adult worm in definitive hosts [5]. The extracellular matrix (ECM)-rich pericyst represents a shell-like “barrier” which at the minimum, restricts cyst growth and may even lead to spontaneous abortion of the parasite [6, 7]. Efficient immune response associated with the fibrotic shell around the metacestode might be a major factor of its limitation to a single cyst and of the difference between the pathological features of *E. granulosus* and *E. multilocularis* metacestodes which are otherwise very similar in their genetic and physiological features [8, 9]. Several studies have been published on the characteristics of fibrosis in alveolar echinococcosis (AE), due to *E. multilocularis* [10]; the role of transforming growth factor β (TGF-β) in host’s tolerance to the metacestode and in the development of fibrosis has also been reported in AE [11]. However, very few studies have addressed these questions in CE.

In diseases characterised by fibrosis, there is excessive scarring caused by production, deposition, and cross-linking of ECM [12]. Hepatic stellate cells (HSC) have been recognised as the main cell type responsible for ECM protein formation during hepatic fibrosis. HSC are located in Disse’s space and store vitamin A under physiological condition. When liver injury occurs, HSCs become activated in response to oxidative stress, growth factors and inflammatory stimuli, which are produced by damaged hepatocytes, resident macrophages (Kupffer cells), infiltrating inflammatory cells and aggregated platelets. The activated cells then change into myofibroblasts which express alpha-smooth muscle actin (α-SMA) [13, 14]. Activated HSC can deposit ECM components including collagen (COL) 1A1 and COL3A1, fiA1 and CO and laminin at the site of local tissue damage. Meanwhile, they can secrete profibrogenic mediators, such as TGF-β, connective tissue growth factor and platelet-derived growth factor, thereby regulating liver fibrogenesis through a complex feed-back loop [15–17].

An approach that may shed light on the mechanisms involved in the development of liver fibrosis in the pericyst of *E. granulosus* metacestode, and has not been used yet for either of *Echinococcus* spp., is to study gene expression regulated by small RNAs, particularly microRNAs (miRNAs) [18, 19]. miRNAs are small, endogenous, non-coding RNAs that interact with the 3′-untranslated region (UTR) of target mRNAs; this interaction can lead to the inhibition of translation or to the promotion of mRNA degradation [20, 21]. With multiple and diverse targets, miRNAs are important in cellular developmental processes including differentiation, migration and proliferation. Many studies have revealed a role for miRNAs in HSC proliferation and myofibroblastic differentiation. For example, miR-335 was shown to influence HSC migration and activation via, at least in part, inhibition of tenascin-C (TNC) expression [22]. It has also been reported that the inhibition of c-myb and rac-1 expression caused by miR-150 and miR-194 was able to inhibit HSC activation [23]. Through its binding to 3′ UTR of collagen and via the transcriptional regulator SP1, induced expression of miR-29b in activated HSC can lead to the inhibition of ECM production [24].

Previous studies have shown that miR-19b, a member, with miR-19a, of the miR-17-92 cluster, can negatively regulate TGF-β signaling components by decreasing TβRII and Smad3 expression [25]. The role of miR-19 in liver fibrosis induced by *E. granulosus* has not been explored. Therefore, in the present study, our aim was to observe the expression of miR-19 in fibrosis liver of CE patients and in activated HSCs, and then to study the function of miR-19 on HSC activation in vitro.

## Methods

### Ethics statement

The clinical investigation was conducted according to the principles expressed in the Declaration of Helsinki. For research involving human participants, informed written consent was obtained from the patients, as part of a research project approved by the Ethical Committee of the First Affiliated Hospital of Xinjiang Medical University (20140812–5).

### Total RNA extraction

Liver biopsy specimens were obtained from 39 patients with CE (Table 1). Each of them was treated by surgery; samples of pericystic tissue and respective adjacent normal liver tissue were taken at operation. The adjacent normal tissue was usually collected at sites 1.5–2 cm away from the parasitic cyst. Total RNA was purified from liver tissue using TRizol reagent (Invitrogen, Carlsbad, CA, USA) and miRNeasy Mini kit (Qiagen, Dusseldorf, Germany) according to the manufacturer’s protocol. The RNA was re-suspended in suitable amounts of DEPC water for further use.

**Table 1:** Baseline characteristics of patients

Characteristics	Patients with CE
Age (years)	34 ± 12
Female sex (%)	29 (59.18)
Stage of CE	active stage/inactive stage (34/15)
ALT (U/l)	20 (5–40)
AST (U/l)	20 (8–40)
Eosinophils	0.46 ± 0.12
Leukocytes (×10^9^/l)	7.07 ± 0.4 (4–10)
Total bilirubin (g/l)	19.05 ± 2.97
Total protein (g/l)	62.12 ± 1.83 (60–83)
Albumin (g/l)	37.58 ± 1.14 (34–48)
Globulin (g/l)	27.89 ± 0.81 (20–35)
ALP (U/l)	63.60 (40–150)
Total bile acid (μmol/l)	10.37 ± 2.13 (0–20)

*Abbreviations*: *ALT* Alanine Amino Transferase, *AST* Aspartate Amino Transferase, *ALP* Alkaline Phosphatas

### Quantitative real-time PCR

Quantitative real-time PCR (QRT-PCR) was performed as described previously [26]. The quantity and quality of the RNA extracted was determined using a nanodrop spectrophotometer (Thermo Fisher Scientific, Waltham, MA, USA). Total RNA (1 μg) was used to synthesize cDNA utilizing miScript Reverse Transcriptase Kit (Qiagen) according to the manufacturer’s protocol and the cDNA amplified was re-suspended in suitable amount of DEPC Water. Then the cDNA samples (2 μl) were used for qRT-PCR in a total volume of 20 μl using the miScript SYBR Green PCR Kit (Qiagen) and miRNA-specific forward primers (Qiagen, Cat. No. MS00031584, Table 2); mi-R19b primers were used in all experiments performed in this study. The amplification profile was as follows: denaturation at 95 °C for 15 min, followed by 40 cycles at 95 °C for 15 s, 55 °C for 30 s and 70 °C for 30 s. All values were normalised to U6 small nuclear RNA (snRNA) (Qiagen, Cat. No. MS00033740, Table 2). For mRNA analysis, total RNA was isolated and cDNA synthesis performed as previously described. The 2^-ΔΔCt^ method was used to calculate relative mRNA expression levels as normalised to β-actin. In all cases, each PCR trial was performed with triple samples and repeated at least three times. The gene-specific oligonucleotide primers (Sangon Biotech, Shanghai, China) are listed in Table 3.

**Table 2:** List of primers used in miScript SYBR Green qRT-PCR

Gene	Accession No.	Product name	Product No.	Cat. No.	Company
hsa-miR-19b-3p	MIMAT0000074	Hs_miR-19b_2 miScript Primer Assay	218300	MS00031584	QIAGEN
*Homo sapiens* RNU6-6P	NG_034215	Hs_RNU6-2_11 miScript Primer Assay	218300	MS00033740	QIAGEN

**Table 3:** List of primers used in SYBR Green qRT-PCR

Gene	Sequence (5′ to 3′)	
*Homo sapiens* COL1A1	Forward	CATGTTCAGCTTTGTGGACCTC
(NM_000088.3)	Reverse	TCACAGATCACGTCATCGCA
*Homo sapiens* COL3A1	Forward	CTTCTCTCCAGCCGAGCTTC
(NM_000090.3)	Reverse	GTAGTCTCACAGCCTTGCGT
*Homo sapiens* TβRII	Forward	GTAGCTCTGATGAGTGCAATGAC
(NM_003242.5)	Reverse	CAGATATGGCAACTCCCAGTG
*Homo sapiens* α-SMA	Forward	CAGCCAAGCACTGTCAGGAAT
(NM_001613.2)	Reverse	TTTGCTCTGTGCTTCGTCAC
*Homo sapiens* β-actin	Forward	TGGCACCCAGCACAATGAA
(NM_001101.3)	Reverse	CTAAGTCATAGTCCGCCTAGAAGCA

### Cell culture and stimulation

The LX-2 cell line was obtained from Shanghai Institutes for Biological Sciences (Shanghai, China). The LX-2 cells were maintained in Dulbecco’s Modified Eagle Medium containing Nutrient Mixture F-12 (DMEM/F-12) (Gibco, Life technologies, New York, USA) supplemented with 10 % Fetal calf serum (Gibco, Life technologies, New York, USA). The cells were incubated at 37 °C at an atmosphere of 5 % CO_2_. For stimulation, the cells were plated on 6 cm dishes starved in medium containing 1 % FCS. The cells were divided into two groups and for the control group, no treatment was performed. For the HCF group, stimulation with HCF for 48 h was carried out.

HCF was obtained by aspiration under aseptic conditions from liver cysts found in clinical CE patients with surgery. The hydatid fluid was centrifuged at 1000 g at 4 °C and the supernatant stored at −80 °C until use. The total protein concentration of HCF was determined using the commercially available bicinchoninic acid (BCA) assay (Thermo Fisher Scientific, Waltham, MA, USA).

### MTT assay

Cell proliferation was determined by standard 3-(4, 5-dimet-hylthiazol-2-yl)-2, 4-diphenyl-tetrazolium bromide (MTT) assay. Briefly, LX-2 cells were placed at a density of 3 × 10^3^ cells per well in 96-well culture plates for 24 h and were then transfected with the miR-19b mimics or negative control mimics using Lipofectamine 2000 (Invitrogen, Carlsbad, CA, USA) according to the manufacturer’s instructions. Cell proliferation was assessed 24, 48, 72 and 96 h. After culturing, 5 mg/ml MTT (Sigma-Aldrich, Louis, MO, USA) was added and incubated at 37 °C for another 4 h. And then, the medium was replaced and the formazan crystals were dissolved in 150 μl dimethyl sulphoxide (DMSO) (Sigma). After 48 h, the medium was changed and the cells were cultured for additional 24–48 h using the MTT assay for cell proliferation measuring. The absorbance was measured at 490 nm using spectrophotometer.

### Cell cycle analysis

For the cell cycle study, PI/RNase Staining Buffer Kit (BD, Biosciences, San Jose, CA, USA) was used. The cells were washed twice with cold PBS, fiThe in 75 % ethanol in PBS and stored at 4 °C overnight. After fiand st, ethanol was removed by centrifugation and the cells were washed twice with cold PBS and stained with 0.5 ml of PI/RNase staining buffer at 37 °C for 30 min in the dark. Analyses were performed on BD LSR flst cytometer (BD, Biosciences, San Jose, CA, USA). The experiments were repeated three times with duplicates for each treatment.

### Transient transfection

For transfection, the cells were placed in DMEM/F12 supplemented with 10 % FCS at a density of 2–3 × 10^5^ cells/ml. The cells were transfected with the miR-19b mimics or the nonspecific (NS)-miRNA (GenePharma, Shanghai, China) using Lipofectamine 2000 (Invitrogen, Carlsbad, CA, USA) according to the manufacturer’s instructions at a final concentration of 50 nM for 24 h. The culturing medium was changed 6 h after transfection and HCF was added at a concentration of 125 μg/ml. The sequences of oligonucleotides used are listed in Table 2.

### Western blot analysis

Proteins were isolated and protein concentration was measured using BCA protein assay. The proteins were subjected to SDS-PAGE electrophoresis and transferred to polyvinylidene fluoride membrane (Millipore Corp, Massachusetts, USA). After blocking, membranes were incubated with primary antibodies (β-actin, Type I Collagen, Type III Collagen, Smad2/3 and TβRII, Santa Cruz Biotechnology, Dallas, Texas, USA; p-smad2/3, Cell Signaling Technology, Danvers, USA) overnight at 4 °C followed by incubation with AP-conjugated secondary antibodies (Cell Signaling Technology, Danvers, USA). The proteins were visualised with BCIP/NBT kit (Invitrogen, Carlsbad, CA, USA).

### Statistical analysis

The Mann-Whitney *U* test was used to analyse the distribution of continuous variables. The Jonckheere-Terpstra test for ordered alternatives was used to identify trends among classes. Correlation coefficients between parameters were evaluated by Spearman’s rank correlations. A two-tailed *P* value < 0.05 was considered significant.

## Results

### Expression of miR-19b is decreased in patients with CE

**Fig. 1:**
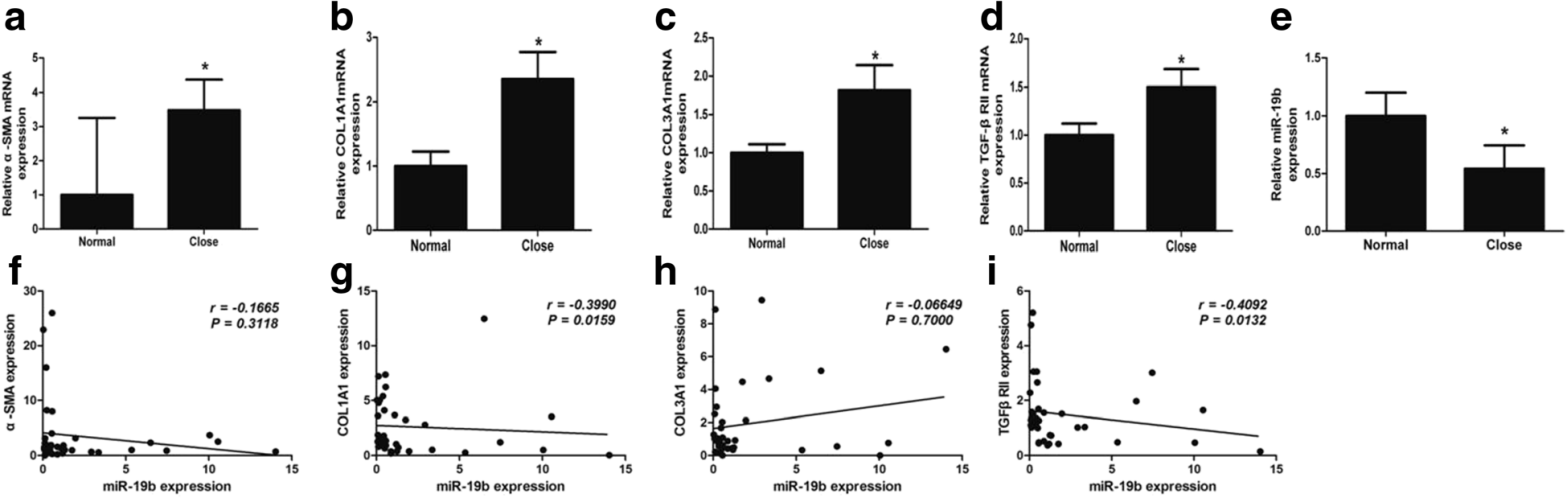
Levels of α-SMA, COL1A1, COL3A1, TβRII mRNA and miR-19b expression in patients with Cystic Echinococcosis (CE) and correlation between α-SMA, COL1A1, COL3A1 or TβRII mRNA expression and miR-19b. **a** Levels of α-SMA mRNA in patients with CE. **b** Levels of COL1A1 mRNA in patients with CE. **c** Levels of COL3A1 mRNA in patients with CE. **d** Levels of TβRII mRNA in patients with CE. **e** Levels of miR-19b in patients with CE. **f** Correlation between miR-19b expression and α-SMA mRNA expression. **g** Correlation between miR-19b expression and COL1A1 mRNA expression. **h** Correlation between miR-19b expression and COL3A1 mRNA expression. **i** Correlation between miR-19b expression and TβRII mRNA expression. Normal: liver parenchyma distant from parasitic cyst; ‘Close’: liver parenchyma close to parasitic cyst. *P < 0.05 *vs* Normal

To assess the progression of liver fibrosis, the expression of fibrosis-related genes was investigated in 39 patients with CE using qRT-PCR; mRNA expression of ECM proteins, COL1A1 and COL3A1 and of α-SMA, were significantly increased. Compared with the adjacent normal liver tissues, miR-19b expression was significantly downregulated in fibrotic liver samples and COL1A1 mRNA expression was significantly negatively correlated with the expression of miR-19b (*r* = −0.3990, *P* = 0.0159) (Fig. 1a-e).

To determine the potential roles of miR-19b in liver fibrosis induced by *E. granulosus*, components of the TGF-β1 signaling pathway that are potential targets were identified by using miRbase Targets and Targetscan 5.1. More attention was paid to TβRII, because TβRII occupies a central role in transduction of TGF-β1/Smad signals. TβRII mRNA was significantly up-regulated and its expression level was significantly inversely correlated with the expression of miR-19b (*r* = −0.4092, *P* = 0.0132) (Fig. 1f-i).

### HCF alters the cell cycle profile of LX-2 cells

**Fig. 2:**
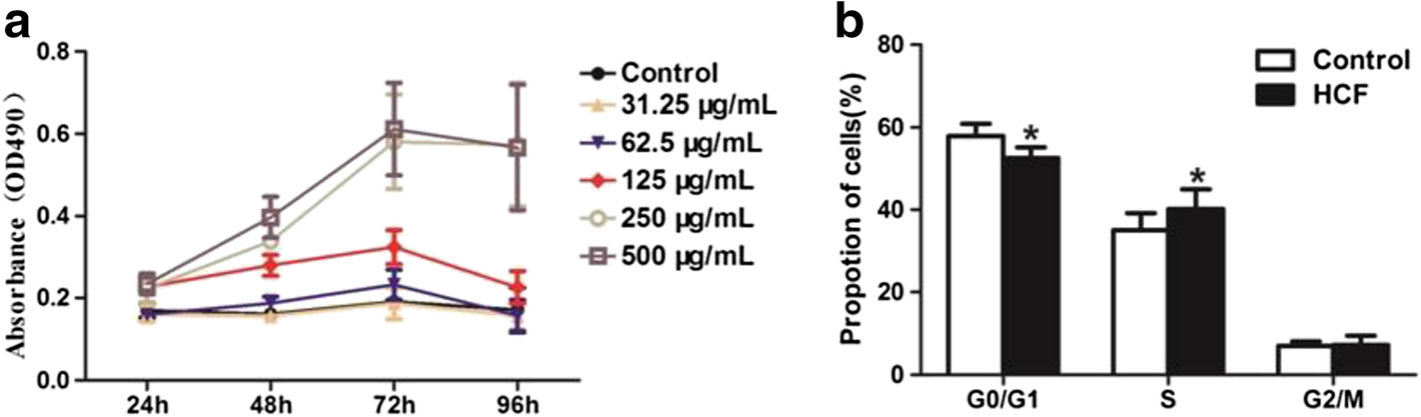
Effect of HCF on LX-2 cells proliferation and cell cycle. **a** The effect of HCF on LX-2 cells proliferation. **b** The effect of HCF on cell cycle in 48 h. Cells were treated with 125 μg/ml HCF for 48 h, as determined by FACS analysis. Control: the non-treated cells. *P < 0.05 *vs* Control

As the data obtained from human livers suggested that parasite components might contribute to the decrease of miR-19b in CE patients, we first performed the MTT assay to assess HSC cell proliferation. Incubation with HCF promoted a significant proliferation of LX-2 cells. As compared to the control cells, cells incubated with HCF differed significantly in their cell cycle distribution, with a lower percentage of cells in the G_1_/G_0_ phase and higher percentage of cells in the S phase (Fig. 2).

### miR-19b is downregulated in HCF-treated LX-2 cells

**Fig. 3:**
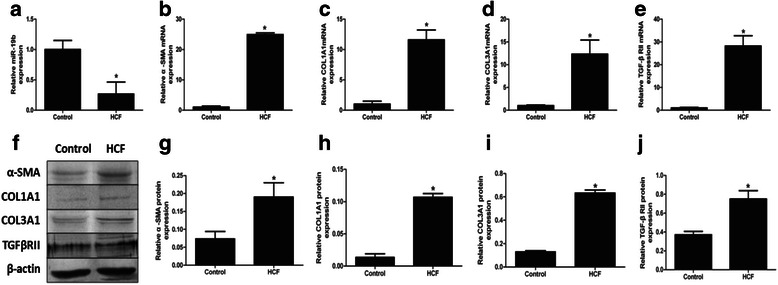
Effect of HCF on the expression of miR-19b, α-SMA, COL1A1, COL3A1 and TβRII in LX-2 cells. **a** Expression of miR-19b in HCF treated LX-2 cells. **b** Expression of α-SMA mRNA in HCF treated LX-2 cells. **c** Expression of COL1A1 mRNA in HCF treated LX-2 cells. **d** Expression of COL3A1 mRNA in HCF treated LX-2 cells. **e** Expression of TβRII mRNA in HCF treated LX-2 cells. **f** Expression of α-SMA, COL1A1, COL3A1 and TβRII protein in HCF treated LX-2 cells for 48 h. **g**–**j** Western blot results were quantified using densitometry analysis. *P < 0.05 *vs* Control

As activation and proliferation of HSC represent key features of liver fibrosis and are characterised by specific gene expression patterns such as high α-SMA and collagen expression, we used qRT-PCR and western blot to assess the expression of such genes. Treatment of LX-2 cells with HCF (125 μg/ml) resulted in the increased expression of α-SMA, COL1A1 and COL3A1. HCF also induced a decrease in the expression of miR-19b. In addition, expression of the target TβRII mRNA and protein was significantly increased after incubation with HCF for 48 h when compared with non-treated cells (Fig. 3).

### miR-19b inhibits HCF-induced LX-2 proliferation

**Fig. 4:**
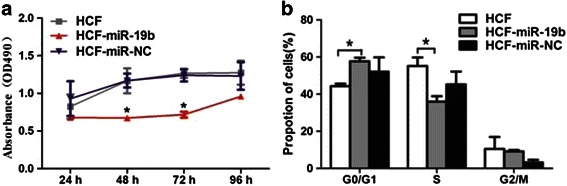
Effect of miR-19b overexpression on the proliferation and cell cycle distribution of HCF-induced LX-2 cells. **a** The effect of miR-19b overexpression on the proliferation of HCF-induced LX-2 cells by MTT assay. **b** The effect of miR-19b on cell cycle of HCF-induced LX-2 cells was analysed by flow cytometry. *P < 0.05

To investigate whether miR-19b was involved in the proliferation of HSCs, miR-19b mimics was transiently transfected into LX-2 cells and we tested the effect of the transfection on the proliferation of HCF-treated LX-2 cells by MTT assay. Overexpression of miR-19b mimics in HCF-treated LX-2 resulted in a significant inhibition of cell proliferation at 72 h, when compared with HCF-treated LX-2 transfected with NS-miRNA. We subsequently analysed the effect of miR-19b mimics on LX-2 cell cycle by flow cytometry. Cell cycle distribution of HCF-treated LX-2 cells transfected with miR-19b mimics differed significantly from the HCF-treated control cells transfected with NS-miRNA, with a lower percentage of cells in the S phases but a higher percentage of cells in the G_2_/M phases. (Fig. 4).

### miR-19b negatively regulates the expression of ECM and TβRII in HCF-induced LX-2 cells

**Fig. 5:**
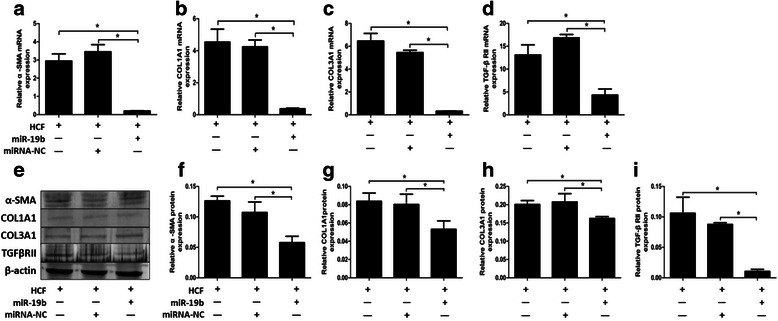
Effect of miR-19b on the expression of α-SMA, COL1A1, COL3A1 and TβRII in LX-2 cells. LX-2 cells were transiently transfected with miR-19b mimics (50 nM) or miRNA negative control (miR-NC) and **a**, α-SMA; **b**, COL1A1; **c**, COL3A1; **d**, TβRII gene expression were assessed by QRT-PCR at 48 h. **e**, 48 h post-transfection cells were harvested and immunoblot performed on whole cell lysates for α-SMA, COL1A1, COL3A1 and TβRII. Relative amount of **f**, α-SMA; **g**, COL1A1; **h**, COL3A1; **i**, TβRII expression were quantified using densitometry analysis. *P < 0.05

To identify the effects of miR-19b overexpression on HCF-induced HSC activation and ECM genes production, we used both qRT-PCR and western blot. Overexpression of miR-19b significantly inhibited α-SMA, COL1A1 and COL3A1 mRNA and protein expression levels in HCF-treated LX-2 cells. Furthermore, overexpression of miR-19b significantly decreased expression of TβRII when compared to cells transfected with NS-miRNA (Fig. 5).

## Discussion

This study is among the rare ones to address a possible influence of components secreted by the metacestode of *E. granulosus* on the pericystic liver fibrosis which surrounds parasite cysts and limits their development. We have shown that, in the liver surrounding the parasitic cyst in CE patients, expression of miR-19 was downregulated and significantly negatively correlated with COL1A1 and TβRII mRNAs. HCF promoted the proliferation of HSC and functioned as a positive regulator during cell transition from G1/G0 phase to the S phase in these cells in vitro. HCF was also able to decrease miR-19 expression in HSC and to increase key-markers of HSC activation, α-SMA, COL1A1 and COL3A1. In addition and conversely, we could show that overexpression of miR-19 inhibited the proliferation of HCF-induced HSC cells by directly targeting TβRII. Taken together, these results suggest that components of parasitic origin present in the cyst fluid are involved in the development of fibrosis through the activation of HSC and that modulation of miR-19 expression is part of the regulatory mechanisms of pericystic fibrogenesis in CE. Although we used miR-19b primers all along our experiments, we propose that, given the high sequence homology between miR-19a and miR-19b and thus the difficulty to estimate which of the two was detected by the SYBR Green PCR, our conclusions conservatively apply to miR-19.

TGF-β is the most potent stimulus for HSC activation and collagen deposition, as it is essential for the differentiation of HSCs into myofibroblasts. As a consequence, inhibition of TGF-β receptors by dominant-negative or soluble TGF-β receptors has been developed and studied in experimental trials in order to reverse fibrosis [27]. Multiple components in the TGF-β signaling pathway have already been characterised as targets of miRNAs [28]. Recent studies have shown the negative regulation of TβRII signaling by members of the miR-17-92 cluster (19a, 19b, 92a), with miR-19b exhibiting the highest fold-change among the cluster members [25]. Li et al. reported that miR-17-92 cluster could inhibit TGF-β pathway and this inhibition would depress proliferation and collagen synthesis in PBMCs by directly targeting TβRII, Smad2 and Smad4 [29]. Moreover, it was shown that delivery of miR-19b into human umbilical vein endothelial cells can block the transition from S phase to the G1/M phase by controlling cyclin D1 expression [30]. The low expression of miR-19 we observed in the pericystic area compared to adjacent normal liver tissues, and its negative correlation with extracellular matrix proteins and TβRII, thus suggest that reduced levels of miR-19 play a role in the pathophysiology of CE. Previous studies, including from our group, have stressed the influence of the TGF-β/Smad activation pathway on the outcome of infections by *Echinococcus* spp. [11, 31], since its impact on the host-parasite interactions encompasses immune tolerance mechanisms, hepatic (and metacestode?) cell proliferation as well as fibrogenesis. We now provide evidence that miR-19 could be a player in its regulation.

The low expression of miR-19, however, might deserve several explanations and have consequences on a variety of cells. Our hypothesis was that it could act, among others, on HSC and it could be induced indirectly by cytokines/growth factors secreted by the cells of the host’s immune response and/or directly by components of *E. granulosus*. HCF is a complex biological mixture that contains a wide range of proteins of both parasite and host origin; it especially contains excretory/secretory proteins produced by the germinal layer (and the protoscoleces) of the metacestode. This is why, after we have shown that miR-19 expression was downregulated in the pericystic liver tissue of patients, we also used HCF and an in vitro model of HSC activation (LX-2 cells) as a first step to study the responsibility of metacestode components in the development and regulation of liver fibrogenesis around the cyst [32, 33]. Based on our study, HCF simulation of LX-2 cells did promote their proliferation as well as the expression of α-SMA and synthesis of COL1A1 and COL3A1, while downregulating miR-19 levels. These findings strongly suggest that HCF play a TGF-β-like fibrosis promoting role in HSC activation and proliferation by influencing miR-19 expression. Wu et al. reported the presence of TGF-β in the periparasitic fibrotic tissue close to hepatic parenchyma in CE patients [34]. Zheng et al. recently reported that *E. granulosus* genome encoded TGF-β-like and TGF-β R-like glycoproteins, which may function in host-parasite crosstalk [35]. Intervention of TGF-β-like proteins of parasitic origin might act in the feedback loop of TGF-β/Smad system regulation, including downregulation of miR-19. However, intervention of other proteins of parasitic origin cannot be excluded.

Fibrosis plays an important role in the overall immune response against several infectious agents, especially those due to viruses, mycobacteria and parasites [36–38], and recent studies have identified a role for miRNAs in the process of inflammatory granuloma formation and subsequent hepatic fibrosis [39]. Lakner et al. found miR-19b levels are downregulated in patients with fibrosis-associated viral infections (HBV and HCV-related chronic hepatitis and hepatocellular carcinoma) and in experimental animal models of hepatic fibrosis [25]. Expressions of miR-146b, miR-155, miR-26b and miR-124 were also shown to be regulated in the mouse liver infected with *Schistosoma japonicum*, *Plasmodium chabaudi* malaria, or *Fasciola gigantica* [40–42]. Fibrosis is an important component of the pathophysiology of each disease caused by *Echinococcus* spp.; however, this role differs markedly in CE and AE. In CE, due to *E. granulosus*, the rapidly established periparasitic fibrosis surrounding the laminated layer likely contributes to the unicystic feature of the disease and to limiting cyst growth [8]; this acellular fibrotic area is used by surgeons as a limit for the dissection of CE cysts to perform cystectomy [43]. In AE, due to *E. multilocularis*, the slow fibrogenesis in an extensive and partially unsuccessful periparasitic granuloma does not prevent germinal layer budding, hence the multi-microcystic ‘alveolar’ feature of the disease; but in the long term it eventually leads to a dense and irreversible fibrosis, responsible for the main complications of the disease, i.e. bile duct and vessel obstruction and secondary biliary cirrhosis [44–46]. It may be noted that, because of its clinical consequences, fibrosis has received much more attention in *E. multilocularis* than in *E. granulosus* infection [45, 47, 48]; little is known on fibrosis in CE. However, until now, such major differences regarding fibrosis between the two diseases have not received any satisfactory explanation. It may be noted that in *E. multilocularis*-infected mice treated with recombinant IL-12 and protected against metacestode growth, effective healing of initial *E. multilocularis* lesions in the ‘protected’ mice was associated with histopathological features similar to those observed in CE [49]. Early development of fibrosis could thus be crucial in the resistance/susceptibility of the hosts to *Echinococcus* spp*.* Our study which provides new insights into the fibrogenesis process and its regulation by miRNAs in CE can serve as a basis for a new line of comparative research between CE and AE to better understand the fibrogenesis process in the two diseases, with the aim of possible therapeutic applications.

## Conclusions

In summary, our results reveal that miR-19 could inhibit the activation of HSCs. These data, together with other published works, suggest miR-19 might suppress TβRII expression. Furthermore, miR-19 was regulated in the process of hepatic fibrosis induced by *E. granulosus* infection and our complementary in vitro experiments suggest that components of *E. granulosus* cyst fluid could be involved in this regulation.
